# DIA label-free proteomic analysis of murine bone-marrow-derived macrophages

**DOI:** 10.1016/j.xpro.2022.101725

**Published:** 2022-09-26

**Authors:** Christa P. Baker, Iain R. Phair, Alejandro J. Brenes, Abdelmadjid Atrih, Dylan G. Ryan, Roland Bruderer, Albena T. Dinkova-Kostova, Douglas J. Lamont, J. Simon C. Arthur, Andrew J.M. Howden

**Affiliations:** 1 School of Life Sciences, University of Dundee, Dow Street, Dundee DD1 5EH, Scotland; 2 Division of Cellular and Systems Medicine, School of Medicine, University of Dundee, Ninewells Hospital and Medical School, James Arrott Drive, Dundee DD1 9SY, Scotland; 3 MRC Mitochondrial Biology Unit, University of Cambridge, Cambridge CB2 0XY, Scotland; 4 Biognosys, Schlieren, Zurich 8952, Switzerland

**Keywords:** Bioinformatics, Immunology, Protein biochemistry, Proteomics, Mass spectrometry

## Abstract

Here, we describe an optimized protocol to analyze murine bone-marrow-derived macrophages using label-free data-independent acquisition (DIA) proteomics. We provide a complete step-by-step protocol describing sample preparation utilizing the S-Trap approach for on-column digestion and peptide purification. We then detail mass spectrometry data acquisition and approaches for data analysis. Single-shot DIA protocols achieve comparable proteomic depth with data-dependent MS approaches without the need for fractionation. This allows for better scaling for large sample numbers with high inter-experimental reproducibility.

For complete details on the use and execution of this protocol, please refer to Ryan et al. (2022).

## Highlights

• Generate deep proteomes without fractionation or labeling
• Tryptic peptides generation from whole cell lysates using the S-Trap assay
• Detailed protocol for DIA-based mass spectrometry
• Data analysis pipeline using Spectronaut and Perseus software packages

## Abstract (editor highlights)

Publisher’s note: Undertaking any experimental protocol requires adherence to local institutional guidelines for laboratory safety and ethics.

## Graphical abstract

**Figure:**
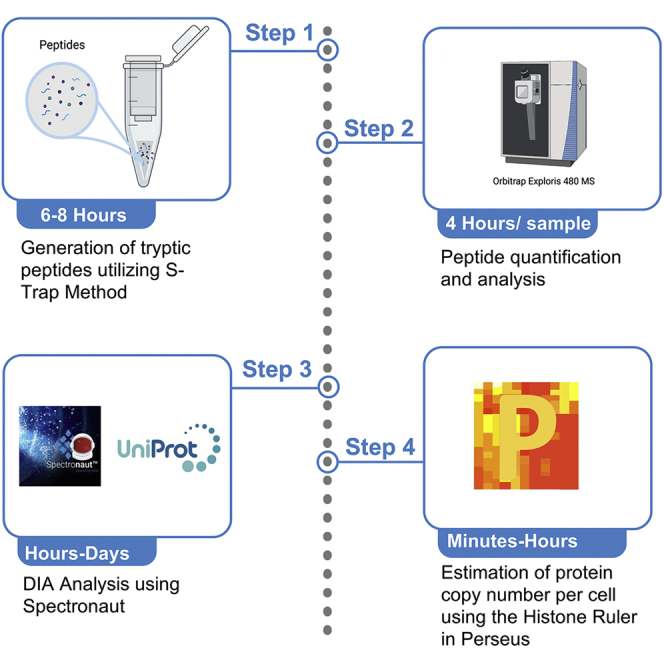

## Before you begin

### Institutional permissions

All animal work was carried out under a UK Home Office license (PAAE38C7B) and approved by the University of Dundee Ethical review and Welfare Committee.

You should ensure that you are compliant with any institutional and national regulations before using this protocol for work involving live vertebrates or higher invertebrates or human clinical samples.

The preparation of bone marrow-derived macrophage (BMDM) lysates for DIA-MS involves several steps. Multiple protocols have been reported for the generation of BMDMs typically using either L929-conditioned media as a source of M-CSF (Toda et al., 2021) or direct supplementation of the media with recombinant M-CSF (Beardmore et al., 2005). Following treatment under desired experimental conditions, cells must be washed thoroughly in PBS to remove serum prior to lysis. Throughout this protocol, it is important to maintain a clean working environment; utilize filter pipette tips and high purity reagents, and wear gloves at all times to prevent contamination during all steps in the process. Additionally, it is important to use disposable plastic-ware instead of beakers for making buffers. Preparation of solutions prior to the S-Trap method (ProtiFi) is important in order to move smoothly through the protocol.

If needed, sample quality and experimental conditions can be validated prior to DIA-MS analysis. In this case, at the point of collecting samples for proteomics, aliquots of cell lysate or culture supernatant can also be collected for use in appropriate assays. Quality control/validation experiments will be experiment dependent; however, could include the analysis of cytokine production by ELISA or activation of signaling by immunoblotting. If the experiment uses a genetic knockout, loss of the protein could be confirmed by immunoblotting or PCR based methods used to confirm the genotypes of the cultures. Cell lysates for proteomic analysis can be stored at −80°C while quality control experiments are performed.

Although we chose to use the S-Trap method for peptide generation, the Single-pot, solid-phase-enhanced sample preparation (SP3) technology (Hughes et al., 2019) could be used as an alternative, especially for cell types with lower cell yields. There are multiple methods for protein digests, however a study comparing digest methods of filter-aided sample analysis (FASP), in-solution digestion and S-Trap spin columns showed that the S-Trap method had the greatest outcome for proteome coverage (Ludwig et al., 2018).

Both data-dependent acquisition (DDA) and DIA mass spectrometry methods can be used to analyze proteomes. Label-free DDA is known to suffer from reduced quantification precision and accuracy as well as increased prevalence of missing values (Stead et al., 2008; Albrecht et al., 2010). This is attributed to the stochastic nature of the fragmentation process which typically selects the top 10–30 most abundant ions within each duty cycle in the mass spectrometry runs. As such label free DDA requires extensive sample fractionation, typically 6–24 fractions, in order to minimize these shortcomings. Labeling methods, such as TMT and SILAC can be used with DDA to provide more accurate and consistent quantification across samples, but still require extensive fractionation to provide reasonable proteome depth and analysis of large numbers of samples by these methods is complex. DIA on the other hand, fragments all ions in a specific m/z window, resulting in more complex spectra, but more reproducible identifications as well as quantification. DIA achieves this without the need of additional fractionation, thus decreasing costs and increasing throughput (Huang et al., 2015), but also slightly decreasing the rate of protein identifications. Due to the reduced costs, scalability and high throughput nature of the approach, DIA is becoming the go-to method for whole proteome analysis.

## Key resources table

**Table:** 

REAGENT or RESOURCE	SOURCE	IDENTIFIER
**Chemicals, peptides, and recombinant proteins**		

DMEM, high glucose, pyruvate	Gibco	41966
Penicillin / Streptomycin	Gibco	15140-122
HEPES Buffer (1 M)	Lonza	BE17-737E
L929-conditioned media	Toda et al. (2021)	∼
2-Mercaptoethanol	Sigma	M6250
Fetal Bovine Serum (South American Origin)	Labtech	FCS-SA/500
Sodium dodecyl sulfate solution, BioUltra, for molecular biology, 20% in H_2_O	Sigma	05030
0.5 M Bond-Breaker™ TCEP Solution, Neutral pH	Thermo Fisher Scientific	77720
1 M Triethylammonium bicarbonate (TEAB)	Thermo Fisher Scientific	90114
Methanol for HPLC, ≥99.9%	Sigma	34860-2.5L-R
HiPerSolv Water for HPLC 2.5 L	VWR	83650.320
Phosphoric acid 49.5%–50.5%	Sigma	93752-250 mL
Ammonium bicarbonate	Sigma	A6141-25G
pH-indicator strips pH 5.0–10.0	Merck Millipore	109533
Benzonase (Novagen), Purity >99%	Merck Millipore	70664-3
Trypsin Gold, Mass Spectrometry Grade	Promega	V5280
Filter pipette tips p2, 20, 200, 1,000	ART	∼
1.5 mL Protein LoBind tubes	Eppendorf	525-0133
Protein LoBind tubes 5.0 mL	Eppendorf	Z768820
Iodoacetamide (IAA)	Sigma	L1149
S-Trap™ mini spin columns	ProtiFi	C02-mini-80
Methanol, Certified AR for Analysis	Fisher Chemical	11976961
Acetic Acid Glacial	Fisher Scientific	10171460
Pierce Acetonitrile, LC-MS Grade	Thermo Fisher Scientific	51101
Formic Acid	Thermo Fisher Scientific	695076-500ML
Trifluoroacetic acid	Sigma	302031
DPBS (1×) Dulbecco’s Phosphate Buffered Saline	Gibco	14190-094

**Critical commercial assays**		

EZQ Protein Quantification Kit	Thermo Fisher Scientific	R33200
CBQCA Protein Quantitation Kit	Invitrogen	C6667

**Experimental models: Organisms/strains**		

6–12 week old male/female C57BL6/J mice	The Jackson Laboratory	664

**Software and algorithms**		

Spectronaut	Biognosys (Bruderer et al., 2015)	∼
Perseus	Tyanova et al., 2016	https://maxquant.net/perseus/
Proteomic Ruler plugin for Perseus	Wiśniewski et al., 2014	https://maxquant.net/perseus_plugins/

**Others**		

Thermomixer comfort 2.0 mL	Eppendorf	5382000031
Nunc™ Cell-Culture Treated 6 well plates	Thermo Fisher Scientific	140675
Centrifuge 5920 R	Eppendorf	5948000060
CLARIOStar Plus microplate reader	BMG LABTECH	∼
BioRuptor sonicator	Diagenode	∼
Evaporation GeneVac	Genevac	EZ- 2 Series
Cap-Locks for 1.5–2 mL Microcentrifuge Tubes	Starlab	I1415-1508
Orbitrap Exploris 480 Mass Spectrometer	Thermo Fisher Scientific	∼
Acclaim™ PepMap™ 100 C18 HPLC Columns, 100 μm × 20 mm, 5 μm, 100 Å	Thermo Fisher Scientific	164946
Acclaim™ PepMap™ 100 C18 HPLC Columns, 75 μm × 500 mm, 2 μm, 100 Å	Thermo Fisher Scientific	164942

## Materials and equipment

### Buffers

**Table:** Proteomic lysis buffer

Stock	Final concentration	Volume needed for 5 mL
20% SDS	5%	1.25 mL
0.5 M TCEP	10 mM	100 μL
1 M TEAB	50 mM	250 μL
HiPerSolv Water for HPLC	∼	3.4 mL

***Note:*** Proteomic lysis buffer should be made fresh each day of use.

**Table:** S-Trap binding buffer

Reagent	Stock	Volume per 60 mL
Methanol for HPLC	99.9%	54 mL
Phosphoric acid	50% in HPLC H_2_O	approx. 390 μL (see note)
Triethylammonium bicarbonate (TEAB)	1 M	6 mL

***Note:*** S-Trap binding buffer should be made fresh each day of use.

***Note:*** Volume of S-Trap binding buffer required: 5,200 μL/sample.

Prepare buffer volume accordingly:

• Prepare S-Trap Binding buffer by first adjusting the pH of the 1 M TEAB to 7 with 50% phosphoric acid. Approximately 65 μL of 50% phosphoric acid is required per mL of TEAB. To check the pH, aliquot a small volume of the pH-adjusted buffer and submerge a pH test strip. Adjust with additional phosphoric acid if required until pH 7 is reached at 25°C.

***Note:*** It may be necessary to add additional 50% phosphoric acid to fully neutralize the pH of TEAB to 7.

***Note:*** A pH strip is used rather than a pH meter so that only a small volume of liquid is used for testing the pH. Obtaining a pH close to 7 is sufficient and accurate enough for a functioning S-Trap buffer.

***Note:*** Effervescence at this step is normal; exercise caution.

• Once the pH has been adjusted to 7, add methanol for a final TEAB concentration of 100 mM.

**Table:** Digest buffer

Reagent	Stock	Mass per 10 mL
Ammonium Bicarbonate	∼	39.75 mg
HiPerSolv Water for HPLC	∼	10 mL

***Note:*** Digest buffer can be stored at 25°C for 1 month or 4°C for 1 year.

Weigh out equivalent mass for 50 mM ammonium bicarbonate in HPLC grade water (the molecular weight of Ammonium bicarbonate is 79.5 g/mol).

**Table:** Trypsin stock solution

Reagent	Stock	Amount for 100 μL
50 mM Acetic Acid	∼	100 μL
Trypsin	∼	100 μg

***Note:*** Trypsin stock solution may be aliquoted and stored at −80°C.

***Note:*** Substitution of Trypsin Gold with another supplier may require a different solvent.

***Note:*** For each sample, you will require 5 μg Trypsin per 100 μg protein. For example, if you have 10 samples and plan to digest 280 μg of protein per sample, you will require 140 μL of reconstituted Trypsin (approximately 2 vials—200 μL of 1 μg/μL Trypsin). Plan to have an extra vial of Trypsin-Gold in the freezer. The calculation of this is discussed during the Trypsin Digest subsection in the S-Trap method.

**Table:** Iodoacetamide (IAA)

Reagent	Stock	Amount for 1 mL
Iodoacetamide (IAA)	∼	92.5 mg
HiPerSolv Water for HPLC	∼	1 mL

***Note:*** IAA should be made fresh immediately before use and is light sensitive.

***Note:*** Methyl methanethiosulfonate (MMTS) may be used as an alkylator substitute for IAA (stock 500 mM MMTS, final concentration 20 mM MMTS).

Prepare 1 mL aliquot of IAA (molecular weight 184.96 g/mol). To make IAA stock add 92.48 mg IAA/mL HPLC water.

**Table:** EZQ rinse buffer

Reagent	Stock	Amount for 1 L
Methanol	99.9%	100 mL
Glacial Acetic Acid	∼	70 mL
Milli-Q Water	∼	830 mL

**Table:** 0.2% formic acid

Reagent	Stock	Amount for 10 mL
Formic Acid	≥96%	20 μL
HiPerSolv Water for HPLC	∼	9.98 mL

***Note:*** 0.2% formic acid can be stored at 25°C for 1 month or 4°C for 1 year.

**Table:** 50% acetonitrile containing 0.2% formic acid

Reagent	Stock	Amount for 10 mL
Acetonitrile (ACN)	>99.9%	5 mL
Formic Acid	≥96%	20 μL
HiPerSolv Water for HPLC	∼	4.88 mL

***Note:*** 50% acetonitrile containing 0.2% formic acid can be stored at 25°C for 1 month or 4°C for 1 year.

**Table:** 1% formic acid

Reagent	Stock	Amount 10 mL
Formic Acid	≥96%	100 μL
HiPerSolv Water for HPLC	∼	9.90 mL

***Note:*** 1% formic acid can be stored at 25°C for 1 month or 4°C for 1 year.

**Table:** Elution buffer A

Reagent	Stock	Amount 200 mL
Formic Acid	≥96%	200 μL
HiPerSolv Water for HPLC	∼	199.8 mL

***Note:*** 1% formic acid can be stored at 25°C for 1 month or 4°C for 1 year.

**Table:** Elution buffer B

Reagent	Stock	Amount 200 mL
Acetonitrile	>99.9%	160 mL
Formic Acid	≥96%	200 μL
HPLC Water	∼	39.8 mL

## Step-by-step method details

**Figure 1:**
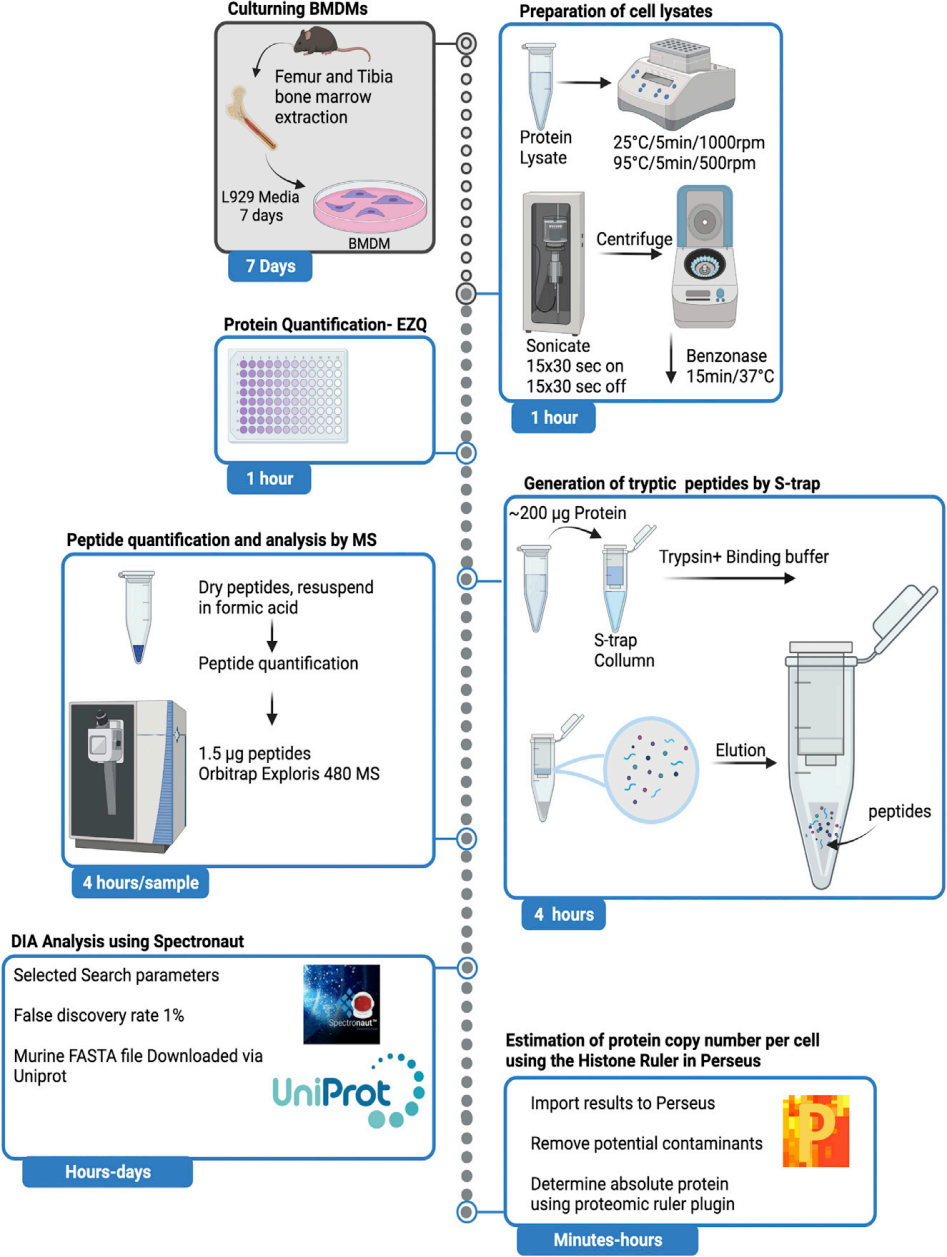
Workflow summary of the main steps in proteomic data acquisition

The overall process is summarized in Figure 1. Each individual step is described in detail below.

### Generation of tryptic peptides utilizing S-Trap method

**Timing: 6–8 h**

This section will provide detailed steps involving the preparation of lysates for tryptic digest and peptide generation. Starting with lysis of bone marrow derived macrophages, lysates are processed and proteins are quantified using the EZQ Method in preparation for the S-Trap Method peptide digest and elution of digest and lastly peptide quantification by CBQCA.

***Note:*** The S-Trap processing method requires a number of centrifugation steps. When deciding how many samples to process, we suggest considering the tube capacity of your microcentrifuge.

Prior to starting the S-Trap method, prepare Proteomic Lysis buffer, S-Trap binding buffer, and Digestion buffer.

1. Differentiate macrophages from mouse bone marrow using established methods (Toda et al., 2021).
2. On day 7, seed macrophages at 1 million cells per well of a 6-well tissue culture plate.
3. On day 8, treat cells under desired experimental conditions.
4. Following treatments, wash cells 3 times using 1 mL of sterile PBS per wash/per well, gently removing the PBS after each wash.

**CRITICAL:** Thorough washing is important to remove all serum from cells prior to lysis.

5. Aspirate PBS entirely and lyse cells in 400 μL of proteomic lysis buffer.

***Note:*** This volume of proteomic lysis buffer is optimal for the specific cell type and seeding density described. The volume of proteomic lysis buffer will require optimization for alternative cell types.

6. Scrape cells and transfer lysates to 1.5 mL LoBind Eppendorf tubes.
7. Place Cap-Locks on tubes to prevent lids popping open during heating.
8. Incubate lysates for 5 min on a ThermoMixer at 95°C with shaking at 500 rpm.

**Pause point:** Cell lysates can be frozen at this point and stored at −80°C to perform quality control or validation experiments on separate lysates. If needed the cell culture media could be collected during step 4, prior to the PBS wash.

9. Incubate lysates for 5 min on a shaker at 25°C with shaking at 1,000 rpm.
10. Place tubes in sonicator and sonicate at maximum amplitude for 15 cycles of 30 s on / 30 s off.

***Note:*** If using a different sonicator than the one described in this protocol, optimization of sonication cycles may be required.

11. Briefly centrifuge tubes up to 4,000 *g* to collect liquid from the lid and sides.

**Optional:** Add 1 μL of benzonase to each sample to degrade DNA and RNA, and incubate for 15 min at 37°C.

***Note:*** Benzonase is an additional and optional step, added to further decrease DNA levels. Although SDS may deactivate Benzonase to a certain extent, we choose to include this step as an additional precaution to limit DNA in lysate, since DNA will bind and block the S-Trap column.

12. Determine protein yield using an EZQ protein quantitation kit (Thermo Fisher Scientific) as per manufacturer’s protocols:
  a. Prepare protein standards of ovalbumin, from the EZQ kit, make 2 mg/mL, 1.0 mg/mL, 0.5 mg/mL, 0.1 mg/mL, 0.05 mg/mL, 0.02 mg/mL and 0.01 mg/mL, using proteomic lysis buffer as the blank.
  b. Mount assay paper on EZQ™ 96-well microplate cassette.
  c. Prepare spots on assay paper mounted on plate (1 μL per well), with 3 replicates per standard and 3 replicates per sample.
  d. Leave to dry.
  e. Transfer the assay paper to a suitable container, add enough methanol to cover the assay paper and incubate for 5 min with shaking at 25°C.
  f. Discard the methanol and leave to dry.
  g. Incubate with 20 mL EZQ stain (enough to cover assay paper) for 30 min with rocking, in the dark (covered in foil).
  h. Discard the solution and add EZQ rinse buffer.
  i. Incubate for 1–2 min with shaking at 25°C.
  j. Repeat steps h and i two more times.
  k. Dry assay paper.
    ***Note:*** Drying steps for washes with rinse buffer can be performed using a hairdryer under a fume hood.
  l. Mount assay paper on the plate.
  m. Analyze using CLARIOStar Plus microplate reader (or equivalent) for protein quantification using excitation/emission settings of ∼485/590 nm.

13. During EZQ incubation step g, add 17 μL of freshly made 0.5 M IAA to each lysate sample after step 11 (optional), to give a final concentration of 20 mM IAA.
14. Incubate samples in the dark at 25°C for 1 h.

**CRITICAL:** IAA is light sensitive, thus this step must be done in the dark.

15. Check the quantity of protein you have from your EZQ assay. If the amount falls in the range of 100–300 μg total protein then you can use the S-Trap mini columns.

***Note:*** If you have more than 300 μg protein, adjust your loading volume so that no more than 300 μg of protein is loaded. Using seeding densities as per step 2, BMDMs yield a surplus of protein, and adjusting the loading volumes to use S-Trap mini columns is sufficient.

***Note:*** If you have less than 100 μg protein, consider using S-Trap micro columns.

***Note:*** If you have many samples you can use the S-Trap 96 well plate.

16. To the lysate from step 15, add 12% aqueous phosphoric acid at 1:10 for a final concentration of 1.2% phosphoric acid (e.g., 40 μL phosphoric acid into 400 μL lysate) and mix briefly by vortexing.

***Note:*** The addition of phosphoric acid is important for the formation of a colloidal protein particulate, prior to loading on the S-Trap column.

17. Transfer each acidified cell lysate to a new 5 mL LoBind Eppendorf tube.
18. Add S-Trap binding buffer at a ratio of 7:1 (v:v) to the acidified SDS lysate.

**Example:** If you have 458 μL acidified SDS lysate, add 3,206 μL S-Trap binding buffer.

19. Mix samples by pipetting up and down.

***Note:*** Colloidal protein particulate is instantly formed in this step. Given sufficient protein, the solution may appear translucent.

20. With the S-Trap mini columns in a 2 mL receiver tube, add the acidified SDS lysate /S-Trap binding buffer mix to the spin column.

***Note:*** Do not add more solution than can fit in the lower, straight portion spin column (650 μL for S-Trap mini columns).

21. Centrifuge the S-Trap mini spin column at 4,000 *g* for 30 s. Protein will be bound and trapped within the protein-trapping matrix of the spin column.

***Note:*** If the acidified SDS lysate/S-Trap binding buffer mix volume is higher than 650 μL, load the column and centrifuge multiple times until the full volume has been bound.

22. Wash captured protein by adding 400 μL S-Trap binding buffer; repeat centrifugation and discard the wash. Repeat this wash step a total of 5 times.
23. Move S-Trap mini spin column to a clean 2 mL sample tube, taking care not to transfer any residual S-Trap binding buffer.
24. Reconstitute trypsin at 1 μg/μL in 50 mM acetic acid.

**Example:** For a 100 μg aliquot of trypsin, add 100 μL of 50 mM acetic acid.

25. From your EZQ calculations, calculate the quantity of trypsin required; for each sample a 1:20 (w/w) ratio of trypsin:sample total protein is used.

**Example:** If 280 μg of protein is loaded onto the S-Trap column, 14 μg of 1 mg/mL trypsin will be required.

26. Prepare a stock digest buffer containing trypsin (125 μL required per sample) and vortex briefly to mix.

**Example:** As per the example above, if 280 μg of protein is loaded onto the S-trap mini column, prepare a trypsin solution containing 14 μL of 1 μg/μL trypsin and 111 μL of Digest Buffer for a total volume of 125 μL per sample.

27. Add 125 μL trypsin solution to the top of each spin column. Centrifuge at 4,000 *g* for 15 s and return any solution that passes through to the top of the column.

***Note:*** The S-Trap mini spin column is highly hydrophilic and will absorb the digestion buffer.

28. Ensure the cap of the S-Trap mini spin column is closed to limit evaporative loss.

***Note:*** The column lid is designed to prevent pressure build up, which would otherwise force the digestion from the protein trap, while limiting evaporative loss.

29. Incubate S-Trap mini spin columns on a ThermoMixer for 2 h at 47°C without shaking, with the heated lid.

***Note:*** This step can be replaced with ThermoMixer overnight 37°C without shaking, with the heated lid. We have not identified any difference in digest efficiency when comparing a 2 h digest with an overnight digest.

**CRITICAL:** Do not shake the columns once the trypsin is added. Shaking will greatly impede performance; some dripping may occur which does not affect performance.

30. After 2 h add 80 μL of digestion buffer (without trypsin) to the S-Trap mini spin column and centrifuge at 1,000 *g* for 60 s. Keep the flow through in the collection tube.

***Note:*** This aqueous elution contains the majority of peptides.

31. This process is repeated with the addition of 80 μL of 0.2% aqueous formic acid to each S-Trap mini spin column and centrifugation at 1,000 *g* for 60 s, collecting the flow through in the same collection tube as step 30.
32. This process is repeated this time with the addition 80 μL of 50% aqueous ACN containing 0.2% formic acid to each S-Trap mini spin column and centrifuge at 4,000 *g* for 60 s for the final elution, collecting the flow through in the same collection tube as steps 30 and 31.

***Note:*** This elution assists in recovery of hydrophobic peptides. Other organics may also be used as desired.

**Pause point:** You can store the eluted peptides at −80°C at this point or continue with the next step.

33. Vacuum dry the samples in an evaporator overnight (8–16 h) at 30°C using a program suitable for aqueous samples (“HPLC” program using the recommended evaporator).
34. After peptides are dried, resuspend in 50 μL 1% formic acid. Pipette up and down to mix.

***Note:*** If you load less protein than the stated range of the S-Trap mini spin column, then consider resuspending in 40 μL of 1% formic acid.

35. Incubate on a ThermoMixer at 30°C at 700 rpm for 1 h.

***Note:*** This step is necessary to fully resolubilize and resuspend peptides.

36. Centrifuge at 20,800 *g* (or maximum speed possible in bench-top centrifuge) for 30 min.
37. Being careful not to disturb the precipitate, pipette liquid into a new 1.5 mL Protein LoBind tube, leaving the precipitate behind as waste.

***Note:*** It is important to leave precipitate behind, since any precipitate can clog the mass spec injector.

38. Perform peptide quantification using the CBQCA quantification kit (Thermo Fisher) as per manufacturer’s protocols.

***Note:*** Peptide quantification is important since there will be loss of some peptides during S-Trap elution.

### Analysis by mass spectrometry

**Timing: 4 h/sample**

In this section, we include detailed settings required for DIA MS run.

39. Analyze 1.5 μg peptide per sample.
  a. Inject the samples onto a nanoscale C18 reverse-phase chromatography system, then electrosprayed into an Orbitrap Exploris 480 Mass Spectrometer. For liquid chromatography buffers are as follows: elution buffer A (0.1% formic acid in HiPerSolv Water (v/v)) and elution buffer B (80% acetonitrile and 0.1% formic acid in HiPerSolv Water (v/v)).
  b. Load at 10 μL/min onto a trap column (100 μm × 2 cm, PepMap nanoViper C18 column, 5 μm, 100 Å, Thermo Scientific) equilibrated in 0.1% trifluoroacetic acid (TFA).
  c. Wash the trap column for 3 min at the same flow rate with 0.1% TFA then switched in-line with a Thermo Scientific, resolving C18 column (75 μm × 50 cm, PepMap RSLC C18 column, 2 μm, 100 Å).
  d. Elute the peptides from the column at a constant flow rate of 300 nL/min with a linear gradient from 3% elution buffer B to 6% elution buffer B in 5 min, then from 6% elution buffer B to 35% elution buffer B in 115 min, and finally to 80% elution buffer B within 7 min.
  e. Wash the column with 80% elution buffer B for 4 min and re-equilibrated in 35% elution buffer B for 5 min. Run two blanks between each sample to reduce carry-over. Keep the column at a constant temperature of 50°C.
40. The data acquisition settings are listed below:

### Data acquisition

**Table:** MS survey scan

Spray source	Positive mode
Spray voltage	2.650 kV
Ion transfer tube Temperature	250°C
MS1 Orbitrap resolution	120,000
MS2 Orbitrap resolution	30,000
Full MS Scan Cycle	350–1,650 m/z
RF Lens (%)	40
AGC (%)	300
maximum injection time mode	Custom
Maximum injection time	20 ms
Source fragmentation	disabled

**Table:** MS survey scan followed by MS/MS DIA Scan events

Multiplex ions	False
Collision energy mode	Stepped
Collision energy type	Normalized
HCD collision energies (%)	25.5,27,30
Orbitrap resolution	30,000
First mass	200
RF lens (%)	40
AGC target	Custom
Normalized AGC target (%)	3,000
Maximum injection time mode	Custom
Maximum injection time	55 ms

***Note:*** If using a different MS instrument, the settings may require optimization.

### DIA data analysis using Spectronaut

**Timing: Hours to days depending on number of raw files to be analyzed**

Spectronaut is a software tool created by Biognosys, a spin-off company of Ruedi Aebersold, one of the principal contributors to the creation of data independent acquisition proteomics. It is currently one of the leading tools for the analysis of Data Independent Acquisition. Overall, it has been shown to provide a robust and reliable performance, coupled with a simpler graphical interface that makes it friendlier to use for non-expert users. This allows users to customize the settings, if they feel knowledgeable enough, or use a pre generated template, if not. It also allows users to customize the reports produced from the DIA data. This is a very valuable feature for non-expert users who want to obtain detailed reports with ease. In short, Spectronaut provides a robust performance and ease of use for non-expert users.

41. Search the raw data using Spectronaut 15 with the direct DIA option against the Mouse C57BL6J Uniprot database (UP000000589, 20210316). Do not use the default parameters. The search parameters are as follows:
  a. Enzyme: Trypsin.
  b. Peptide minimum length: 7 amino acids.
  c. Missed cleavages: 2.
  d. Protein Qvalue Cut-off (Run) should be set to 0.01.
  e. Major Group Top N: unselected, the default value of 3 is incompatible with the “proteomic ruler” method.
  f. Data MS1 and MS2 collected in profile mode.
    **CRITICAL:** The top N parameter must not be enabled as it restricts the number of precursor ions and peptides used to calculate intensities. By default, Spectronaut uses the 3 most abundant ions and the 3 most abundant peptides only. As a result data are lost for proteins where more than 3 peptides are identified. The Proteomic Ruler method used to calculate estimated copy numbers requires all histone intensity to be used, therefore the Top N parameter is incompatible with this method. The ‘Major Group Quantity’ should be set to ‘Sum peptide quantity.’
  g. Minor Group Top N: unselected, the default for 3 is incompatible for reasons stated above.
  h. The ‘Minor Group Quantity’ should be set to ‘Sum precursor quantity’.
  i. ‘Protein LFQ Method’ must be changed to ‘QUANT 2.0 (SN Standard)’.
  j. Cross Run Normalization should be unselected.
  k. Calibration mode: Automatic.
  l. Fixed modifications: Carbamidomethylation of cysteine.
  m. Variable modifications: Oxidation of methionine, deamidation of asparagine and glutamine and acetylation (protein N-terminus).
  n. False discovery rate (FDR) of precursor and protein: 1% Q-value.
  o. Data filtering should be set to ‘Q-value’ to avoid values being imputed.
  p. Filter out decoy hits.
    i. This is done when generating the report on Spectronaut. Within the report tick no decoy in Spectronaut to reverse hits.
  q. Filter out potential contaminants (keratin, trypsin) once the search is complete:
    i. Download database of contaminants from MaxQuant (http://www.coxdocs.org/doku.php?id=maxquant:start_downloads.htm).
    ii. Use the Gene ID to manually remove proteins where the gene name is included in the contaminants list from your dataset.
      ***Note:*** Version 15.4.210913.50606 of Spectronaut was used for this paper; however, the default search parameters must be substantially modified to ensure compatibility with the proteomic ruler method.
      ***Note:*** UniProt Mouse C57BL6J proteome database FASTA file (UP000000589) is available for download at http://www.uniprot.org/proteomes/.
      ***Note:*** Other software can be used instead of Spectronaut such as Skyline and DIA-NN both of which are free. Links are as follows: https://skyline.ms/project/home/software/Skyline/begin.view, https://github.com/vdemichev/diann.

### Estimation of protein copy number per cell using the proteomic ruler in perseus

**Timing: Minutes to hours**

The proteomic ruler is a computational tool for calculating the number of copies per cell of each protein without the need for spiking in standards (Wiśniewski et al., 2014). This method is available as a plugin within the Perseus software package (Tyanova et al., 2016). It uses the relationship between DNA mass and histone mass and correlates it to histone intensity detected in the proteomic experiment to estimate the copy numbers for all proteins detected in the sample. The proteomic ruler plugin can automatically estimate protein copy numbers as well as protein concentration. Requirements of the proteomic ruler include a deep proteome from a eukaryotic nucleated cell with a known ploidy. Accuracy of the histone ruler also requires complete cell lysis with no cellular fractions lost (chromatin required), and at least 12,000 peptides should be identified.

42. Download and install Perseus software.

***Note:*** Perseus software can be freely download at https://maxquant.net/perseus/.

***Note:*** Perseus version 1.6.15.0 was used.

43. Download and install the Proteomic Ruler plugin compatible with your version of Perseus.

***Note:*** The Proteomic Ruler plugin compatible with your version can be freely downloaded at http://www.coxdocs.org/doku.php?id=perseus:user:plugins:store with guidance at http://www.coxdocs.org/doku.php?id=perseus:user:plugins:proteomicruler.

44. Prior to loading the Spectronaut output file (∗.tsv file generated by Spectronaut), adjust the ‘PG.MolecularWeight’ column to ensure that each protein is annotated with a single molecular weight.
  a. Open the ∗.tsv file generated by Spectronaut in Microsoft Excel.
  b. Copy the ‘PG.MolecularWeight’ column onto a new sheet.
  c. Select Data>Text to Columns.
  d. Under ‘Original data type’ select ‘Delimited’.
  e. Under ‘Delimiters’ select ‘Semicolon’.
  f. Under ‘Column data format’ select ‘General’.
  g. Open the ‘Go to’ menu by pressing F5 and select ‘Special’.
  h. Select ‘Constants’ and ensure only ‘Text’ is checked.
  i. Right-click on one of the highlighted cells, and select Delete>Shift cells left.
  j. Ensure every row has a molecular weight value.
  k. Copy the first column from this sheet containing molecular weight values for every protein identified, and paste it over the ‘PG.MolecularWeight’ column on the Spectronaut output file.
  l. Save the Spectronaut output file and close Microsoft Excel.
45. Select ‘Generic Matrix Upload’ (green arrow) and import the Spectronaut output file into Perseus for data processing.
46. From the Spectronaut analysis you will input protein identification, intensities and peptide numbers into Perseus.
47. Determine absolute protein quantification using the Proteomic Ruler plugin (Wiśniewski et al., 2014).
  a. Select Proteomic ruler>Estimate copy numbers and concentrations.
  b. Proteins IDs: PG.ProteinAccessions.
  c. Intensities: Select columns containing intensity values.
  d. Logarithmized: Unchecked.
  e. Averaging mode: All columns separately.
  f. Molecular masses: PG.MolecularWeight.
  g. Detectability correction: Unchecked.
  h. Scaling mode: Histone proteomic ruler.
  i. Ploidy: 2.
  j. Output: Copy number per cell, Concentration [nM], Separate sample summary tab (total protein, total molecules, cell volume…).
48. Add gene ontology (GO) terms and KEGG pathway annotations to each protein in the resultant matrix:
  a. Select Annot. Column>Add annotation.
  b. Source: mainAnnot.mus_musculus.txt.gz.
  c. Uniprot column: PG.ProteinAccessions.
  d. Annotations to be added: GOBP name, GOMF name, GOCC name, KEGG name.

***Note:*** Information for downloading and installing annotation files are found at http://www.coxdocs.org/doku.php?id=perseus:user:use_cases:modifications#add_annotation.

49. Determine the statistical significance of differentially expressed proteins by performing Student’s *t*-test on log-transformed data, and create a volcano plot (scatter plot of all datapoints example in Figure 2D) based on -log_10_[*p*(Student's *t*-test)] against log_2_[fold change].

**Figure 2:**
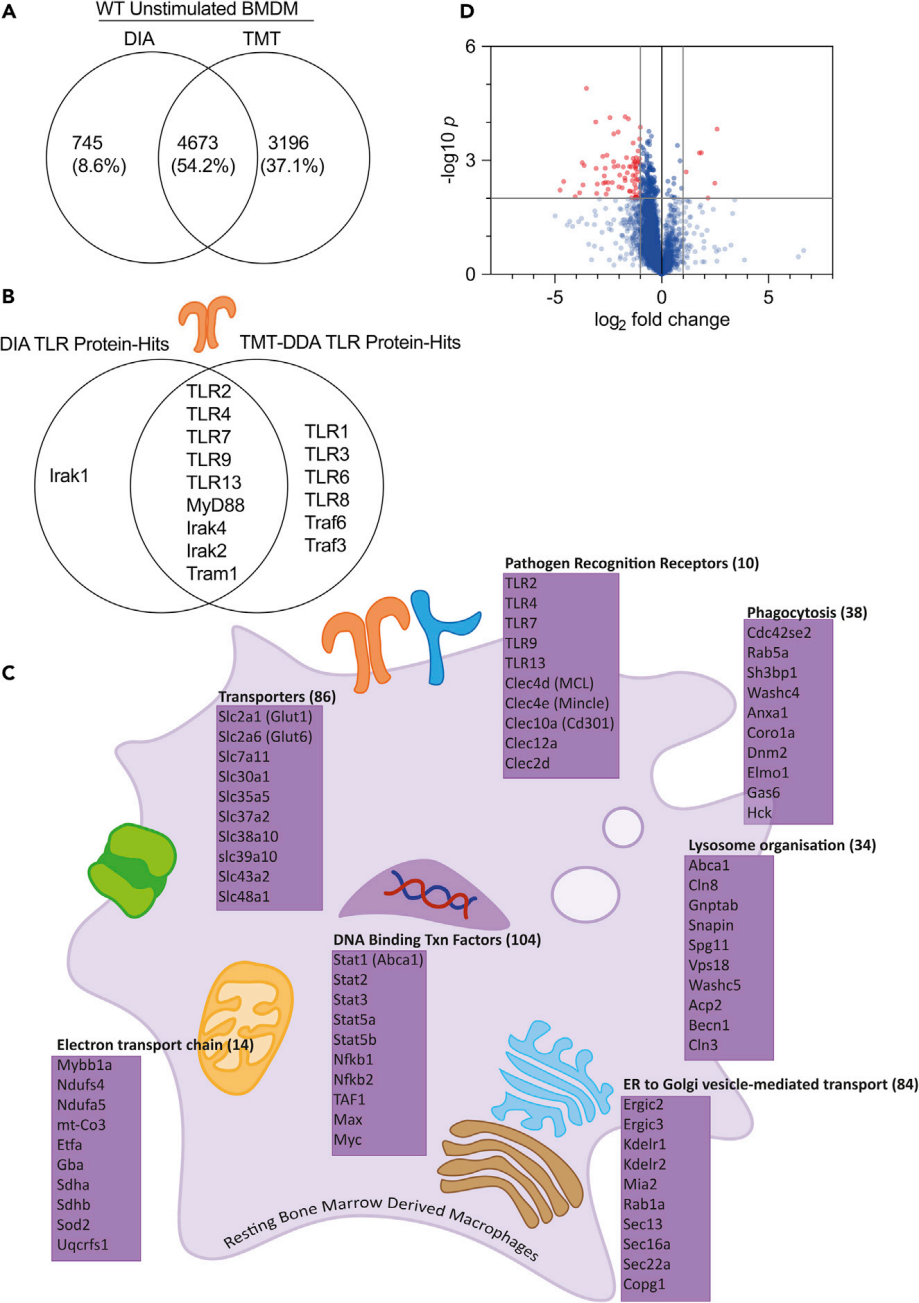
Expected outcomes for DIA proteomic analysis (A–C) Comparisons of the data obtained using DIA and TMT-DDA proteomic methods for unstimulated bone marrow derived macrophages. (A) Venn Diagram comparing DIA and TMT total protein hits. (B) Comparison of the detection of Toll-like receptors (TLRs) between DIA and TMT-DDA datasets. (C) Schematic diagram showing potentially relevant examples of the proteins identified in the DIA dataset, displaying the total number of hits in each cellular component in parentheses, sorted by function using the DAVID Bioinformatics Database. (D) Shows an example volcano plot comparing the proteome of macrophages from nuclear factor erythroid 2-related factor 2 knockout mice (Nrf2 KO) and macrophages from mice with a knockdown of its negative regulator Kelch-like ECH-associated protein 1 Keap1 knockdown (Keap1 KD) obtained using DIA methods. X axis shows the of Log2 fold change in estimated copy numbers, and y axis the -Log10 p-value (Student's *t-*test, n=4). Both datasets have been published previously in (Ryan et al., 2022) and are available in PRIDE (accession number PXD030455).
50. Analyze differentially expressed proteins using freely available bioinformatics tools:
  a. GO enrichment: DAVID (https://david.ncifcrf.gov).
  b. Pathway enrichment: KEGG (https://www.genome.jp/kegg) and Reactome (https://reactome.org).
  c. Transcription factor enrichment: Enrichr (https://maayanlab.cloud/Enrichr/) and ChEA3 (https://maayanlab.cloud/chea3/).

## Expected outcomes

In a previous study (Ryan et al., 2022) the macrophage proteome was analyzed utilizing DIA-MS and TMT-DDA-MS. Here we compare the DIA and TMT proteome coverage from this study. In this study DIA-MS produced a dataset with 5418 proteins identified, of which 4673 proteins were identified in the TMT dataset (Figure 2A) (Ryan et al., 2022). Although identification of 5418 proteins provides reasonable proteome coverage, this is on the low end of what can be expected for DIA-MS. Typically we have achieved between 5,000–7,000 protein identifications for DIA-MS from immune cells. Currently, for studies with small sample numbers, TMT proteomics is considered the gold-standard for depth of protein identification. Although, the depth of coverage of the TMT dataset is greater, with ∼7,000–8,000 proteins identified, this coverage required fractionation of samples into 16, increasing the cost (with fractionation and labeling) of the experiment and reducing the throughput. Additionally, multi-batch TMT datasets are challenging to compare between batches, due to false positives and missing values (Brenes et al., 2019). Alternatively, the single-shot DIA method, achieves considerable depth of proteome coverage without the need for fractionation, and allows better scaling for large sample numbers with high interexperimental reproducibility (Vowinckel et al., 2018; Muntel et al., 2019). An example of relevant proteome coverage and depth can be seen with Toll-like receptor (TLR) proteins, where DIA achieved similar identification outputs as the TMT dataset (Figure 2B). An alternative approach is to use DIA with a library-based search utilizing a previous DDA and/or DIA dataset. This method of analysis may increase peptide identification, providing a dataset with greater depth (Isaksson et al., 2022). Given that many researchers will not have access to a deep proteome library, we have analyzed this data set using the library free direct DIA option to demonstrate the depth of coverage that is possible without the use of a library. In summary, DIA-MS is an effective technique, which provides an in depth look into the changing proteins in macrophages; including but not limited to PRRs, transporters, transcriptions factors as exemplified in Figure 2C.

## Limitations

Using this protocol, the impact of various stimuli on global protein abundance in macrophages can be analyzed, allowing for the absolute quantification of ∼5,500–6,500 proteins. However, not all proteins are identified by this method, and if a higher resolution proteome is required, fractionation of samples may be required. While the method can be used to robustly quantify protein expression levels it does not provide any information on post-translational modifications.

## Troubleshooting

Validation will be experiment dependent. If the experiment relates to inhibition or activation of signaling pathways using a genetic model and/or small molecule inhibitors/activators, you might consider changes in signaling networks e.g., western blot. If conditions used have previously been shown to induce changes in functional responses (e.g., cytokine/chemokine production and/or marker expression) consider using ELISAs or flow cytometry to validate your treatments.

If you have many samples consider using the S-Trap 96 well plate.

S-Trap troubleshooting.

### Problem 1

Precipitate in lysate prior to using S-Trap mini column.

### Potential solution

If starting from frozen lysate samples, and after the sonication step, there is precipitate in lysate, heat the samples for 10 min at 70°C to dissolve any SDS precipitate and then centrifuge at max speed for 30 min and pipette liquid into a new Eppendorf, leaving precipitate behind. This is important, because if there is precipitate in the lysate, this can clog the S-Trap mini column, and prevent adequate peptide elution.

### Problem 2

No or poor peptides captured in the eluate.

### Potential solution

• SDS is necessary for efficient lysis (step 5) and capture of the protein-trapping matrix by S-Trap mini columns. Ensure applied samples contain 5% SDS (see proteomic lysis buffer preparation).
• Make sure the SDS solubilized protein sample is acidified to a final concentration of ∼1.2% phosphoric acid (see S-Trap binding buffer preparation).
• Ensure complete transfer of the acidified lysate/S-Trap buffer solution to the S-Trap mini column, including any particulate or colloid (step 20).
• Ensure amount of protein added is between 100–300 μg. S-Trap mini columns are optimal for 100–300 μg of protein. If you have less material, consider using S-Trap micro columns. If you have more material, consider using S-Trap midi columns (step 15).
• Try different weight/weight concentrations of trypsin, such as increasing the amount of trypsin added (use a 1:10; trypsin: protein) (step 25).
• Try different digestion temperatures/times, such as an overnight digest at 37°C (step 29).
• Ensure entire S-Trap mini column is exposed to heat during digestion by keeping heated lid on (step 29).
• Ensure no bubbles are present during digestion by visual inspection (steps 28/29).
• Assess if digestion is incomplete by monitoring via gel electrophoresis as well as mass spectrometry analysis (post-step 39 analysis).

## Resource availability

### Lead contact

Further information and requests for resources and reagents should be directed to and will be fulfilled by the lead contact, J. Simon C. Arthur (j.s.c.arthur@dundee.ac.uk).

### Materials availability

No new material generated.

## Data Availability

Not applicable.
